# The utility of accessibility clauses in resident contracts

**DOI:** 10.1186/s12909-023-04905-x

**Published:** 2023-11-30

**Authors:** Quinten K. Clarke

**Affiliations:** Department of Psychiatry, University of British Columbia, Vancouver, Canada

**Keywords:** Disability, Accessibility, Resident physicians, Union, EDI, Medical education

## Abstract

Resident organizations and unions have a powerful role in advocating for resident physicians with disabilities. Ongoing efforts to ensure accessibility for resident physicians with disabilities would be promoted through the inclusion of clauses in resident contracts that ensure accessible work environments.

## Text

Accessibility for resident physicians with disabilities is lacking in current medical and academic facilities. Employers have exhibited an inadequate response to addressing the accessibility barriers in their organizations.

The Mass General Brigham Housestaff Union was recently successful in becoming the first resident union in the United States. On June 8, 2023, the vote passed by a margin of 1,215 to 412 [1]. Various other groups across the United States are currently working to unionize their resident staff as a means of collectively advocating for improved working conditions.

In the Canadian context, provincial resident organizations have existed for decades. Resident physicians are automatically members of these organizations which hold the responsibility for negotiating with the relevant provincial government on resident contracts which outline pay, benefits and employer responsibilities. While these organizations are not formally unions, they have many of the same responsibilities and functions.

Resident physicians with disabilities currently navigate educational and occupational environments with varying levels of accessibility that are not built to standards of universal design. When these environments do not meet their accessibility needs, they must appeal to their institution to implement changes or accommodations. If their institution refuses to provide these supports, they must use laborious and slow legal mechanisms such as filing an Americans with Disabilities Act (ADA) complaint or a Human Right Tribunal complaint to appeal such decisions. These legal mechanisms can take several years to resolve making them impractical and contributing to delays in completion of residency for students who require specific accommodations to continue their studies.

In ongoing advocacy efforts related to equity, diversity, inclusion, and accessibility, resident organizations and unions can be powerful advocates. Through their influence over resident contracts, they have the opportunity to negotiate for inclusion of clauses that promote accessibility. Unfortunately, resident organizations have not exercised this influence.

Previous studies have shown that the prevalence of physicians and medical trainees with disabilities is increasing [2–7]. This increased prevalence of physicians and medical trainees with disabilities has occurred in the absence of meaningful changes to the accessibility of work and learning environments, and policies related to employment of physicians and medical trainees with disabilities. As such, physicians and medical trainees with disabilities have entered an environment in which they are not well supported. As noted by Jarus et al. [8]., medical trainees with disabilities are still challenged to negotiate a sense of legitimacy and belonging in medical education settings. This is in part attributable to inaccessible learning environments such as staff-facing hospital spaces including call rooms, examination/interview rooms, and staff parking. When the learning environment is not accessible, trainees with disabilities feel devalued and experience an additional burden in completing their training compared to their non-disabled peers [8].

An accessibility clause included in a residency contract or union collective agreement would formally enshrine the existing right to an accessible work environment. This would further encourage employers of resident physicians to provide accommodations or modifications to resident work environments that would allow those with disabilities to continue their employment. These accessibility clauses would not affirm new rights but rather reaffirm existing rights in the Canadian and American contexts. The Americans with Disabilities Act, and the Canadian Charter of Rights and Freedoms, have both affirmed that medical learners have the right to reasonable accommodations during their training [9–11].

Unfortunately, these rights are often not respected by educational institutions leading resident physicians to use legal means to obtain needed accommodations or accessibility features. These legal mechanisms can take months to years to reach resolution during which time the medical learner may experience psychological distress secondary to having their career disrupted, and losing potential earnings.

By including this clause in the resident contract, it would preclude the need for the involvement of these other legal forums and would allow for internal resolution of such disputes. It will give trainees voice and promote advocacy to support those with disabilities. Further, by including this clause, it would provide an additional protection to the accommodation needs of this equity-seeking group.

It is worth noting that the accessibility clause would provide benefit for those experiencing temporary disability such as a physician recovering from an acute fracture. Ideally, the inclusion of this clause would further encourage medical educators and hospital leaders to consider the principles of universal design when developing or redeveloping teaching hospital environments. By incorporating principles of universal design, and planning for the inclusion of resident with disabilities, fewer trainees would require accommodations and thus the administrative burden would be reduced for medical schools and residency training programs.

## Conclusions

By including these rights in a resident contract, it would allow resident unions and organizations to enforce the contract through refusal of work. Given that much of academic medicine would not function effectively without the additional labour of resident physicians, we anticipate that this would lead to a more rapid transition to accessible environments and universal design than we have previously seen in these spaces using existing Canadian and American disability legislation.

## Data Availability

Not applicable.
